# Exome Sequencing–Based Screening for *BRCA1/2* Expected Pathogenic Variants Among Adult Biobank Participants

**DOI:** 10.1001/jamanetworkopen.2018.2140

**Published:** 2018-09-21

**Authors:** Kandamurugu Manickam, Adam H. Buchanan, Marci L. B. Schwartz, Miranda L. G. Hallquist, Janet L. Williams, Alanna Kulchak Rahm, Heather Rocha, Juliann M. Savatt, Alyson E. Evans, Loren M. Butry, Amanda L. Lazzeri, D’Andra M. Lindbuchler, Carroll N. Flansburg, Rosemary Leeming, Victor G. Vogel, Matthew S. Lebo, Heather M. Mason-Suares, Derick C. Hoskinson, Noura S. Abul-Husn, Frederick E. Dewey, John D. Overton, Jeffrey G. Reid, Aris Baras, Huntington F. Willard, Cara Z. McCormick, Sarath B. Krishnamurthy, Dustin N. Hartzel, Korey A. Kost, Daniel R. Lavage, Amy C. Sturm, Lauren R. Frisbie, T. Nate Person, Raghu P. Metpally, Monica A. Giovanni, Lacy E. Lowry, Joseph B. Leader, Marylyn D. Ritchie, David J. Carey, Anne E. Justice, H. Lester Kirchner, W. Andrew Faucett, Marc S. Williams, David H. Ledbetter, Michael F. Murray

**Affiliations:** 1 Molecular and Human Genetics Department, Nationwide Children’s Hospital, Columbus, Ohio; 2 Genomic Medicine Institute, Geisinger, Danville, Pennsylvania; 3 Laboratory for Molecular Medicine, Partners HealthCare, Cambridge, Massachusetts; 4 Regeneron Genetics Center, Tarrytown, New York; 5 Center for Translational Bioinformatics, University of Pennsylvania, Philadelphia; 6 Department of Genetics, Yale School of Medicine, New Haven, Connecticut

## Abstract

**Importance:**

Detection of disease-associated variants in the *BRCA1* and *BRCA2* (*BRCA1/2*) genes allows for cancer prevention and early diagnosis in high-risk individuals.

**Objectives:**

To identify pathogenic and likely pathogenic (P/LP) *BRCA1/2* variants in an unselected research cohort, and to characterize the features associated with P/LP variants.

**Design, Setting, and Participants:**

This is a cross-sectional study of adult volunteers (n = 50 726) who underwent exome sequencing at a single health care system (Geisinger Health System, Danville, Pennsylvania) from January 1, 2014, to March 1, 2016. Participants are part of the DiscovEHR cohort and were identified through the Geisinger MyCode Community Health Initiative. They consented to a research protocol that included sequencing and return of actionable test results. Clinical data from electronic health records and clinical visits were correlated with variants. Comparisons were made between those with (cases) and those without (controls) P/LP variants in *BRCA1/2*.

**Main Outcomes:**

Prevalence of P/LP *BRCA1/2* variants in cohort, proportion of variant carriers not previously ascertained through clinical testing, and personal and family history of relevant cancers among *BRCA1/2* variant carriers and noncarriers.

**Results:**

Of the 50 726 health system patients who underwent exome sequencing, 50 459 (99.5%) had no expected pathogenic *BRCA1/2* variants and 267 (0.5%) were *BRCA1/2* carriers. Of the 267 cases (148 [55.4%] were women and 119 [44.6%] were men with a mean [range] age of 58.9 [23-90] years), 183 (68.5%) received clinically confirmed results in their electronic health record. Among the 267 participants with P/LP *BRCA1/2* variants, 219 (82.0%) had no prior clinical testing, 95 (35.6%) had *BRCA1* variants, and 172 (64.4%) had *BRCA2* variants. Syndromic cancer diagnoses were present in 11 (47.8%) of the 23 deceased *BRCA1/2* carriers and in 56 (20.9%) of all 267 *BRCA1/2* carriers. Among women, 31 (20.9%) of 148 variant carriers had a personal history of breast cancer, compared with 1554 (5.2%) of 29 880 noncarriers (odds ratio [OR], 5.95; 95% CI, 3.88-9.13; *P* < .001). Ovarian cancer history was present in 15 (10.1%) of 148 variant carriers and in 195 (0.6%) of 29 880 variant noncarriers (OR, 18.30; 95% CI, 10.48-31.4; *P* < .001). Among 89 *BRCA1/2* carriers without prior testing but with comprehensive personal and family history data, 44 (49.4%) did not meet published guidelines for clinical testing.

**Conclusions and Relevance:**

This study found that compared with previous clinical care, exome sequencing–based screening identified 5 times as many individuals with P/LP *BRCA1/2* variants. These findings suggest that genomic screening may identify *BRCA1/2*-associated cancer risk that might otherwise remain undetected within health care systems and may provide opportunities to reduce morbidity and mortality in patients.

## Key Points

**Question:**

Can population-level genomic screening identify those at risk for disease?

**Findings:**

In this cross-sectional study of an unselected population cohort of 50 726 adults who underwent exome sequencing, pathogenic and likely pathogenic *BRCA1* and *BRCA2* variants were found in a higher proportion of patients than was previously reported.

**Meaning:**

Current methods to identify *BRCA1/2* variant carriers may not be sufficient as a screening tool; population genomic screening for hereditary breast and ovarian cancer may better identify patients at high risk and provide an intervention opportunity to reduce mortality and morbidity.

## Teaser

This cross-sectional study investigates the ascertainment of pathogenic and likely pathogenic *BRCA1/2* variants among US adults enrolled in the MyCode Community Health Initiative.

## Introduction

Pathogenic variants in the *BRCA1* (OMIM 113705) and *BRCA2* (OMIM 600185) genes were first associated with familial breast cancer risk in the mid-1990s.^1,2^ Associated risks for ovarian cancer, prostate cancer, pancreatic cancer, and melanoma have since been documented; these clinical associations are grouped as hereditary breast and ovarian cancer (HBOC) syndrome.^3^

Clinical testing for pathogenic variants in the *BRCA1* and *BRCA2* (ie, *BRCA1/2*) gene became available in 1995.^4^ Risk-assessment strategies, based on personal and family history cancer thresholds, have been built, validated, and incorporated into clinical testing guidelines. These guidelines are routinely used to identify individuals with increased pretest probability of pathogenic *BRCA1/2* variants.^5,6^ Current established guidelines have been issued by the National Comprehensive Cancer Network (NCCN),^7^ the US Preventive Services Task Force,^8^ and the American College of Medical Genetics and Genomics together with the National Society of Genetic Counselors.^9^

Clinical underascertainment of individuals with pathogenic *BRCA1/2* variants may be associated with the failure to apply testing guideline criteria and the failure of criteria-based strategies to identify all true positives.^10,11^ Such underascertainment has been documented even in women with existing cancer diagnoses^12,13^ and has prompted calls for DNA sequence–based population screening.^14^

In this article, we report the pathogenic variants prevalence, HBOC syndrome–associated cancer history, and comparison of exome sequencing–based screening with indication-based ascertainment in a cross-sectional study of predominantly white (European ancestry) adults who underwent exome sequencing at a single US health care system (Geisinger Health System, Danville, Pennsylvania) from January 1, 2014, to March 1, 2016. These individuals had “expected pathogenic” (ie, known pathogenic and likely pathogenic) *BRCA1/2* variants identified by exome sequencing. Sequencing-based screening findings were ascertained independent of participants’ age, sex, or cancer history.^15^ The cases and controls are part of the ongoing DiscovEHR cohort and were identified through the Geisinger MyCode Community Health Initiative. This large exome-sequenced research cohort has a median of 14 years of linked electronic health records (EHRs), and the patients consented to a research protocol that included sequencing and return of actionable test results.^15,16^ The genomic findings were confirmed by clinical laboratory testing and entered into the EHR.

## Methods

This cross-sectional study received approval from Geisinger Institutional Review Board. Written informed consent was obtained from participants. This study followed the Strengthening the Reporting of Observational Studies in Epidemiology (STROBE) reporting guideline.

### Cases and Controls Defined

*Cases* are defined as individuals with a pathogenic or likely pathogenic (P/LP) variant in either the *BRCA1* or *BRCA2* gene. We referred to these cases as *BRCA1/2* carriers to be consistent with published literature. *Controls* are defined as the participants who underwent sequencing in the biobank (MyCode; Geisinger) and screened negative for P/LP variants in both the *BRCA1* and *BRCA2* genes. We further subdivided the case population on the basis of (1) prior identification of a variant, (2) personal or family history that would have met established clinical criteria for referral or testing, and (3) gene involvement.

### HBOC Syndrome Diagnosis and Relevant Cancers

For the purposes of this article, an HBOC syndrome diagnosis was assigned in cases in which both genotype and phenotype were present. Therefore, HBOC syndrome diagnosis refers to cases in which a relevant cancer occurred (ie, *BRCA1/2* carriers whose genetic risk was associated with personal history of relevant cancer at any age). The relevant cancers included breast, ovarian, prostate, pancreatic, and melanoma.

### Participant Consenting and Study Oversight

The biobank and the associated clinical return of results were approved by the Geisinger Institutional Review Board.^16^ Enrollment in the biobank by written informed consent began on January 1, 2007. Participants who consented prior to October 1, 2013, did not explicitly agree to have their genomic results returned to their health care practitioner and entered into the EHR; these were considered outdated consents and not eligible for clinical result return if efforts to reconsent were unsuccessful. Individuals with outdated consents are included in the biobank’s data analysis.

### Participants, Whole Exome Sequencing, and EHR Data

Analysis for this article was limited to 50 726 health system patients who underwent exome sequencing as participants in the biobank, aged 18 years or older, with EHR data, and with whole exome sequencing data available through the DiscovEHR study.^15^ Additional details on the biobank study design, EHR data extraction, and sequenced data characteristics have been previously described.^15,16^

The *BRCA1/2* carrier subgroup who had undergone prior clinical *BRCA1/2* testing was significantly younger and had an overrepresentation of women when compared with *BRCA1/2* carriers without prior testing (Table 1).

**Table 1.** Participant and Subgroup Demographics

Variable	Active Health System Patient (n = 1 253 024)	Biobank Participant		Subgroup of Cases					
		Without *BRCA1/2* Variant (Control) (n = 50 459)	With *BRCA1/2* Variant (Case) (n = 267)	Vital Status		Testing Status		*BRCA1* vs *BRCA2* Case	
				Deceased, With *BRCA1/2* Variant (n = 23)	Living, With *BRCA1/2* Variant (n = 244)	With Prior *BRCA1/2* Test (n = 48)	Without Prior *BRCA1/2* Test (n = 219)	With *BRCA1* Variant (n = 95)	With *BRCA2* Variant (n = 172)
Age, median (range), y	50.1 (18-89)	59.9 (18-89)	58.9 (23-90)	64.2 (41-87)	58.4 (23-90)	54.9 (28-81)	59.8 (23-90)	57.0 (24-87)	59.9 (23-90)
Women, No. (%) [age range, y]	668 483 (53.4) [18-89]	29 880 (59.2) [18-89]	148 (55.4) [23-90]	12 (52.2) [41-81]	136 (55.7) [23-90]	36 (75.0) [28-77]	112 (51.1) [23-90]	52 (54.7) [25-87]	96 (55.8) [23-90]
Men, No. (%) [age range, y]	584 451 (46.6) [18-89]	20 579 (40.8) [18-89]	119 (44.6) [24-89]	11 (47.8) [51-87]	108 (44.8) [24-89]	12 (25) [42-81]	107 (48.9) [24-89]	43 (45.3) [24-87]	76 (44.2) [27-89]
Self-identified race/ethnicity, No. (%)									
White	1 159 920 (92.6)	49 623 (98.3)	265 (99.2)	23 (100)	24 (9.8)	48 (100)	217 (99.1)	95 (100)	170 (98.8)
African American	46 728 (3.7)	549 (1.1)	2 (0.7)	0	2 (0.8)	0	2 (0.9)	0	2 (1.2)
Other	46 376 (3.7)	287 (0.6)	0	0	0	0	0	0	0
Latino/Hispanic	39 756 (3.2)	546 (1.1)	3 (1.1)	1 (4.3)	2 (0.8)	0	3 (1.4)	1 (1.1)	2 (1.2)
Jewish	6131 (0.5)	148 (0.3)	0	0	0	0	0	0	0

### Bioinformatic Interpretation of Whole Exome Sequencing and Sanger Confirmation

The American College of Medical Genetics and Genomics criteria for variant classification was established to more uniformly categorize variants on the basis of standardized criteria.^17^ Using conservative interpretation to reduce false-positives, we identified *BRCA1/2* carriers with expected pathogenic variants on the basis of (1) classification in ClinVar with a *2 or *3 status, indicating strong evidence for pathogenicity^18^; (2) predicted loss of function; or (3) both (eTable 1 in the Supplement). For identified cases, we sent a DNA sample to the Clinical Laboratory Improvement Amendments–certified Laboratory for Molecular Medicine at Partners HealthCare for clinical variant interpretation and Sanger confirmation prior to clinical result return. Benign variants and variants of unknown significance were not returned. All confirmed P/LP variants are deposited in ClinVar per Laboratory for Molecular Medicine protocol.

We used identity by descent analysis to determine if relatedness contributed to observed prevalence (eTable 2 in the Supplement).^19^ For high-level analysis, we compared data, including demographic information and cancer diagnoses, with the EHR data of the general health system population, but the primary analysis involved a comparison of cases and controls from the biobank population.

### Diagnostic Disposition

Primarily, HBOC syndrome refers to patients who have a relevant cancer and identification as P/LP *BRCA1/*2 carrier, but in the case of screening (both familial carrier testing and population level), this application is not sufficient. Therefore, we employed a previously described diagnostic framework for incidental or secondary genomic findings. The framework’s 5 diagnostic groups include 3 groups with relevant cancer diagnoses (ie, clinical findings consistent with the genomic risk result are present) and in which the conventional diagnosis of HBOC is made as well as 2 groups in which relevant clinical findings are absent in the participants (Table 2). Diagnostic disposition was assigned after a clinical evaluation, when possible, associated with the return of results; alternatively, EHR data were used.

**Table 2.** Diagnostic Disposition of *BRCA1/2* Cases^a^

HBOC Syndrome Diagnosis	No. (%)								
	Case With *BRCA1/2* Variant (n = 267)	Subgroup of Cases							
		Sex		Vital Status		Testing Status		*BRCA1* vs *BRCA2* Case	
		Women With *BRCA1/2* Variant (n = 148)	Men With *BRCA1/2* Variant (n = 119)	Deceased, With *BRCA1/2* Variant (n = 23)	Living, With *BRCA1/2* Variant (n = 244)	With Prior *BRCA1/2* Test (n = 48)	Without Prior *BRCA1/2* Test (n = 219)	With *BRCA1* Variant (n = 95)	With *BRCA2* Variant (n = 172)
Diagnosis^b^									
Groups 1-3	52 (19.5)	39 (26.4)	13 (10.9)	11 (47.8)	41 (16.8)	14 (29.2)	38 (17.4)	30 (31.6)	22 (12.8)
Group 1	14 (5.2)	13 (8.8)	1 (0.8)	4 (17.4)	10 (4.1)	14 (29.2)	NA	13 (13.7)	1 (0.6)
Group 2	34 (12.7)	23 (15.5)	11 (9.2)	7 (30.4)	27 (11.1)	NA	34 (15.5)	16 (16.8)	18 (10.5)
Group 3	4 (1.5)	3 (2.0)	1 (0.8)	NA	4 (1.6)	NA	4 (1.8)	1 (1.1)	3 (1.7)
No diagnosis^b^									
Groups 4-5	215 (80.5)	109 (73.6)	106 (89.1)	12 (52.2)	203 (83.2)	34 (70.8)	181 (82.6)	65 (68.4)	150 (87.2)

Abbreviations: HBOC, hereditary breast and ovarian cancer; NA, not applicable.

^a^ The diagnostic groupings for incidental or secondary genomic findings^20^ were applied as follows: group 1 = prior clinical testing and personal history of disease, group 2 = no prior clinical testing and personal history of disease, group 3 = no prior clinical testing and new diagnosis of disease identified in the initial evaluation after result disclosure, and groups 4-5 = no prior clinical testing and no disease at the time of disclosure (group 4 is the subset that will develop disease subsequently). Significant differences in HBOC syndrome diagnoses were seen between women and men (odds ratio [OR], 2.92; 95% CI, 1.47-5.77; *P* = .002), deceased and living (OR, 4.54; 95% CI, 1.87-10.99; *P* = .001), prior testing and no prior testing (OR, 2.37; 95% CI, 1.13-4.99; *P* = .02, and *BRCA1* and *BRCA2* genes (OR, 3.15; 95% CI, 1.69-5.86; *P* = .002). The ORs and *P* values were calculated after controlling for age, given the substantial age difference between the prior-testing and no-prior-testing groups in Table 1.

^b^ An HBOC syndrome diagnosis is based on an individual with both a personal history of relevant cancer and a pathogenic and likely pathogenic *BRCA1/2* variant. This diagnosis was achieved in groups 1, 2, and 3.

### Clinical Result Return and Family History Assessment

**Figure 1.**
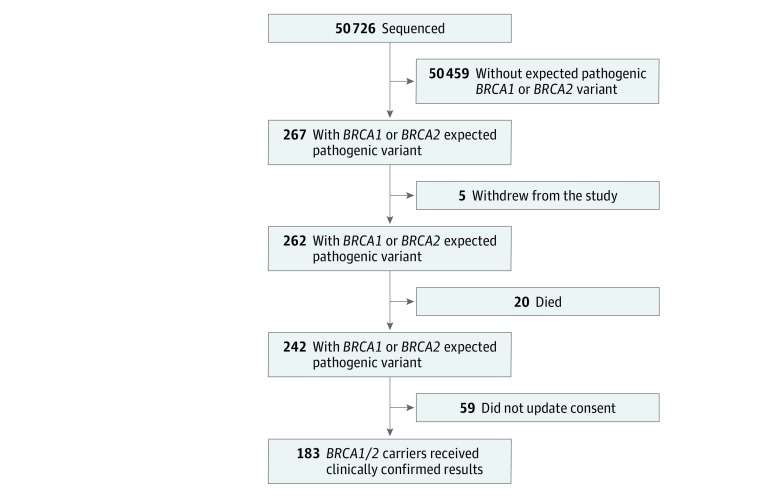
Participant Eligibility for Return of Results Of the 267 *BRCA1/2* carriers, 183 (68.5%) received the clinically confirmed result through an electronic health record portal and were offered clinical risk management. In total, 84 participants withdrew from the study, died, or did not update their consent; these cases had only deidentified records and were not eligible for returned results.

Clinical laboratory reports were entered into the EHR of 183 of 267 (68.5%) *BRCA1/2* carriers (Figure 1) between May 1, 2015 and June 30, 2017. These participants were then (1) supported to pursue NCCN guidelines–based clinical management^7,21^ and (2) encouraged to complete a targeted 3-generation family history (either in person or online). Each pedigree was independently analyzed by at least 2 licensed genetic counselors (A.H.B., M.L.B.S., M.L.G.H., J.L.W., A.K.R., H.R., and J.M.S.), who assessed threshold criteria for referral and testing according to the guidelines of the NCCN,^7^ US Preventive Services Task Force,^8^ and American College of Medical Genetics and Genomics–National Society of Genetic Counselors.^9^ Discordance between the reviewers was resolved by joint review and consensus. A total of 122 relevant 3-generation pedigrees were analyzed, of which 89 (72.9%) were from participants who had not undergone prior *BRCA1/2* clinical testing (Table 3).

**Table 3.** Analysis of Referral and Testing Criteria in 122 *BRCA1/2* Cases

Variable	No. (%)														
	Case With P/LP *BRCA1/2* Variant and Completed Pedigrees (n = 122)^a^			Subgroup of Cases											
				Testing Status						*BRCA1* vs *BRCA2* Case					
				With Prior *BRCA1/2* Test (n = 33)			Without Prior *BRCA1/2* Test (n = 89)			With *BRCA1* Variant (n = 44)			With *BRCA2* Variant (n = 78)		
	All (n = 122)	Women (n = 70)	Men (n = 52)	All (n = 33)	Women (n = 24)	Men (n = 9)	All (n = 89)	Women (n = 46)	Men (n = 43)	All (n = 44)	Women (n = 24)	Men (n = 20)	All (n = 78)	Women (n = 46)	Men (n = 32)
Age, median (range), y	58.6 (23-89)	53.8 (23-87)	65.1 (39-89)	55.2 (28-81)	49.5 (28-71)	70.3 (57-81)	59.9 (23-89)	56.1 (23-87)	64.0 (39-89)	56.9 (28-87)	49.7 (28-71)	65.6 (39-87)	59.6 (23-89)	56.0 (23-87)	64.8 (47-89)
Meeting NCCN testing guidelines	77 (63.1)	51 (72.9)	26 (50.0)	32 (96.9)	23 (95.8)	9 (100)	45 (50.5)	28 (60.9)	17 (39.5)	34 (77.2)	22 (91.7)	12 (60.0)	43 (55.1)	29 (63.0)	14 (43.8)
Meeting NCCN referral guidelines	80 (65.6)	53 (75.7)	27 (51.9)	32 (96.9)	23 (95.8)	9 (100)	48 (53.9)	30 (65.2)	18 (41.9)	34 (77.2)	22 (91.7)	12 (60.0)	46 (58.9)	31 (67.4)	15 (46.9)
Meeting ACMG-NSGC referral guidelines	70 (57.4)	47 (67.1)	23 (44.2)	31 (93.9)	22 (91.7)	9 (100)	39 (43.8)	25 (54.3)	14 (32.6)	33 (75)	21 (87.5)	12 (60.0)	37 (47.4)	26 (56.5)	11 (34.4)
Meeting USPSTF referral criteria^b^	NA	28/49 (57.1)	NA	NA	12/14 (85.7)	NA	NA	16/35 (45.7)	NA	NA	9/13 (69.2)	NA	NA	19/36 (52.8)	NA
BRCAPRO-calculated *BRCA1/2* mutation risk >10%^c^	30 (24.6)	20 (28.6)	10 (19.2)	19 (57.6)	14 (58.3)	5 (55.5)	11 (12.4)	6 (13.0)	5 (11.6)	19 (43.2)	13 (54.2)	6 (30.0)	11 (14.1)	7 (15.2)	4 (12.5)

Abbreviations: ACMG-NSGC, American College of Medical Genetics and Genomics–National Society of Genetic Counselors; NA, not applicable (denotes those who could not be evaluated using these criteria); NCCN, National Comprehensive Cancer Network; P/LP, pathogenic and likely pathogenic; USPSTF, US Preventive Services Task Force.

^a^ This analysis included only the 122 cases in which comprehensive personal and family history data were available. Among those without prior testing, 51% (45 of 89) met NCCN testing criteria; this included 61% of women (28 of 46) and 40% of men (17 of 43).

^b^ The USPSTF referral criteria are applicable only to women and those without a personal history of cancer.

^c^ BRCAPRO calculates pretest probability for *BRCA1/2* testing.^6^

### Deceased Participants

Twenty participants died before their result became available, and 3 died after the return of result (before June 30, 2017). Medical record review was carried out by 3 clinicians (K.M., D.M.L., and M.F.M.) independently and then jointly to determine the cancer diagnosis and cause of death (eTable 3 in the Supplement).

### Statistical Analysis

Data were summarized using means and ranges for continuous variables as well as frequencies and percentages for categorical variables. Comparisons between groups were performed using Wilcoxon rank sum test, Pearson χ^2^ test, and 2-sided Fisher exact test, as appropriate. Logistic regression was used to estimate odds ratios (ORs) and 95% CIs. Two-sided *P* < .05 was considered statistically significant. All analyses were performed using SAS, version 9.4 (SAS Institute Inc).

## Results

Of the 50 726 health system patients who underwent whole exome sequencing, 50 459 (99.5%) had no expected pathogenic *BRCA1/2* variants and 267 (0.5%) were *BRCA1/2* carriers. Of the 267 cases (148 [55.4%] were women and 119 [44.6%] were men with a mean [range] age of 58.9 [23-90] years), 183 (68.5%) received clinically confirmed results in their EHR. Compared with the health system’s overall population, the biobank cohort was older (mean [range] age, 50.1 [18-89] years vs 59.9 [18-89] years; *P* < .001), had a greater percentage of women (668 483 [53.4%] vs 29 880 [59.2%]; *P* < .001), had a higher representation of white patients (1 159 920 [92.6%] vs 49 623 [98.3%]; *P* < .001), and was enriched for relevant cancers (862 [1.7%] vs 1254 [0.10%]; *P* < .001).

### Expected Pathogenic *BRCA1/2* Variants

The prevalence of participants with a Sanger sequencing–confirmed P/LP *BRCA1/2* variants was 1:190, and 1:180 when controlling for relatedness (eTable 2 in the Supplement). Ninety-five patients (35.6%) were identified with *BRCA1* variants, and 172 patients (64.4%) had *BRCA2* variants. There were 118 P/LP variants in *BRCA1/2* in the 267 carriers (eTable 1 in the Supplement). Of the 118 variants, 113 (95.8%) were previously classified in ClinVar: 103 P/LP with a *2 or *3 status,^18^ 5 P/LP by a single laboratory, 1 variant of unknown significance, and 4 with conflicting interpretation entries. Pathogenic or likely pathogenic vs variant of unknown significance interpretations agreed with 99.1% (108 of 109) of the existing clinical significance designations in ClinVar; the single difference was 1 listed in ClinVar as variant of unknown significance that was designated as LP on the basis of updated information.

Of the 52 *BRCA1* and 66 *BRCA2* variants, 107 (90.67%) were putative loss-of-function variants (66 frameshift, 31 nonsense, and 10 splice variants) and only 5 (4.2%) were novel predicted loss-of-function variants. Eleven of 118 variants (9.3%) were missense, and no novel missense variants were classified as P/LP, in accordance with the criteria set forth by the American College of Medical Genetics and Genomics for variants interpretation.^17^

Sanger sequencing confirmed 267 bioinformatically identified *BRCA1/2* carriers and ruled out 1 candidate carrier as a false-positive. The number of *BRCA1/2* carriers with any given variant ranged from 1 to 24 (eTable 1 in the Supplement). Most variants (74 of 118 [62.7%]) were only seen in 1 carrier. Two well-documented common US *BRCA2* pathogenic variants (ClinVar variation ID: 38082 and 9320)^2,22^ accounted for 42 *BRCA1/2* carriers, and 1 of 3 Ashkenazi Jewish founder mutations was found in 19 *BRCA1/2* carriers.^3^

### Personal or Family History of Syndromic Cancers

**Figure 2.**
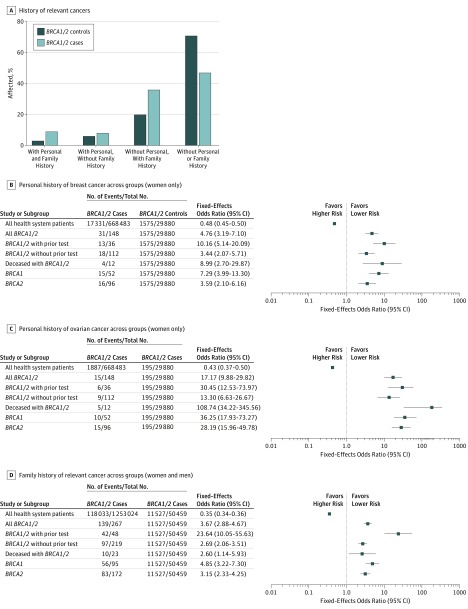
Association of Pathogenic and Likely Pathogenic *BRCA1/2* Variants With Relevant Syndromic Cancers A, Relevant cancers in this analysis included breast, ovarian, prostate, pancreatic, and melanoma. *BRCA1/2* controls included individuals without pathogenic and likely pathogenic *BRCA1/2* variants. *BRCA1/2* cases included *BRCA1/2* carriers. Health system patients also included those who did not undergo screening. Mantel-Haenszel test was used to determine the fixed-effects odds ratio.

Forty-eight (17.9%) of the 267 *BRCA1/2* carriers were already aware of their result through prior clinical *BRCA1/2* testing; these were patients who were diagnosed on the basis of traditional detection methods such as personal and family history. Newly diagnosed *BRCA1/2* carriers (without prior clinical testing) were more likely than controls (without *BRCA1/2* variants) to have EHR evidence of personal or family history of HBOC syndrome–associated cancer diagnoses (28.0% vs 53.4%) (Figure 2A and eTable 5 in the Supplement). New carriers were significantly more likely to have had breast cancer, ovarian cancer, and family history of any HBOC syndrome–associated cancer (Figure 2B-D). In 219 (82.0%) *BRCA1/2* carriers without prior clinical *BRCA1/2* testing compared with controls, the OR for personal history of any HBOC syndrome–associated cancer was 2.32 (95% CI, 1.63-3.29; *P* < .001), indicating significantly increased cancer risk associated with the *BRCA1/2* carriers ascertained in this fashion.

Syndromic cancer diagnoses were present in 11 (47.8%) of the 23 deceased *BRCA1/2* carriers and in 56 (20.9%) of all 267 *BRCA1/2* carriers. Among women, 31 (20.9%) of 148 variant carriers had a personal history of breast cancer, compared with 1554 (5.2%) of 29 880 noncarriers (OR, 5.95; 95% CI, 3.88-9.13; *P* < .001). Ovarian cancer history was present in 15 (10.1%) of 148 variant carriers and in 195 (0.6%) of 29 880 variant noncarriers (OR, 18.30; 95% CI, 10.48-31.4; *P* < .001).

### Return of Results and Extended Family History 

Primarily because of outdated consent, not all participants were eligible to receive results (Figure 1). Of 267 *BRCA1/2* carriers, results were returned to 183 eligible adults (68.5%) and their health care practitioner through a secure EHR portal; this was followed up by a letter and telephone call to the participants. Clinical care based on NCCN guidelines^7^ was offered, and 3 examples of early cancer detection were described in a report.^21^ A case of a woman in her 40s with subclinical disease detected on screening was identified after that report (eFigure 1 in the Supplement).

Analysis of the pedigrees of the 122 carriers on whom we had sufficient personal and family cancer history is found in Table 3. Among the criteria-based assessment tools, the NCCN criteria appeared to be the most sensitive, with 63% of pedigrees meeting the guidelines for testing. Of the 33 individuals who had prior testing, 32 (96.9%) met the NCCN criteria; of the 89 individuals who had not had prior testing, 45 (50.5%) met the criteria and 44 (49.4%) did not meet the criteria. Across all carriers, 34 (77.2%) of 44 *BRCA1* carriers and 43 (55.1%) of 78 *BRCA2* carriers met the NCCN criteria. In nearly every category, women were more likely than men to meet criteria thresholds (Table 3) on the basis of relevant cancer diagnosis (22 of 70 [31.4%] had personal history); underreporting of family health history in men appears to be a contributing factor in this observation (eFigure 2 in the Supplement).^23^

### Diagnostic Dispositions and Cause of Death Analysis

The participants’ final diagnostic category was based on all relevant clinical information reviewed, including the EHR and participant self-report when available (Table 2). The HBOC syndrome diagnosis, assigned in individuals with both P/LP variants and a relevant cancer, constitutes groups 1, 2, and 3 (Table 2) using the groupings for incidental or secondary genomic findings.^20^ The diagnosis was made in 52 (19.5%) of all cases (39 [26.4%] of women; 13 [10.9%] of men).

Twenty-three (8.6%) of 267 participants were deceased by June 1, 2017, and the median age at death was 64.7 years (eTable 3 in the Supplement). Twenty participants died prior to the return of results, and 3 died after the return of results. The observed lifetime occurrence of any relevant cancers in the 23 deceased participants was 47.8% (75.0% in women; 18.2% in men). Among the 23 deaths, the cause of death was determined to be *BRCA1/2*-associated cancer in 9 people (39.1%), unrelated to *BRCA1/2* in 11 (47.8%), and indeterminate in 3 (13.0%). In contrast to the deceased cases, 45 (18.4%) of 244 living patients have a personal history of a relevant cancer (eTables 3 and 4 in the Supplement).

## Discussion

Findings demonstrate that routine exome sequencing–based screening may be a potential factor in long-term care improvement. This study found that only 17.9% of *BRCA1/2* carriers had prior *BRCA1/2* clinical genetic testing in the course of their ongoing health care. In addition, in the subcohort of 89 individuals without prior testing but with detailed personal and family history data, 45 (50.5%) met current NCCN criteria for testing but had not received testing; conversely, 44 individuals (49.4%) did not meet NCCN testing criteria but had expected pathogenic variants. Of importance, those patients who did not meet the established thresholds for *BRCA1/2* testing were not spared from relevant cancers, as evidenced by the diagnosis of early asymptomatic cancer in such individuals after the initiation of cancer risk management.^21^

Our evidence demonstrates that this clinical underascertainment may be associated with both the failure to apply criteria-based referral for *BRCA1/2* testing strategies to individuals with high pretest probabilities and the failure of such criteria-based strategies to be sufficiently sensitive to identify all true positives. The practical difficulties of incorporating sufficient family history risk analysis in routine health care settings are well described.^24^ The observed gap in relevant cancer history between all living *BRCA1/2* carriers and deceased *BRCA1/2* carriers signals an opportunity to prospectively identify and manage individuals with elevated risk to reduce the morbidity and mortality associated with their defined cancer risk. Early success in this area is demonstrated by the identification of 4 individuals with early-stage cancer during the screening and management recommended by the exome sequencing–based screening results process.

Our findings show considerable clinical underascertainment of individuals with HBOC syndrome. Underascertainment has also been observed in familial hypercholesterolemia and Lynch syndrome, 2 conditions that (along with HBOC syndrome) are designated as tier 1 (ie, disease with available evidence-based guidelines and recommendations that potentially positively affect public health) by the Centers for Disease Control and Prevention Office of Public Health Genomics and are included in the Healthy People 2020 recommendations.^25,26^ Underascertainment of these conditions, in which presymptomatic case identification is advantageous,^21,25^ should prompt prioritization of further research into genomic sequencing–based population screening.^27^

Overall, ascertainment through sequencing-based screening in the biobank cohort showed an empirical prevalence for *BRCA1/2* expected pathogenic variants of 1:190, adjusted to 1:180 when controlling for relatedness up to the third degree.^19,25^ Consistent with other studies, HBOC syndrome–associated cancer rates were higher in women than in men and in *BRCA1* carriers than in *BRCA2* carriers.^3,28^ Unsurprisingly, those with prior clinical *BRCA1/2* testing also had higher cancer rates than those without prior testing. For the subset of 23 deceased *BRCA1/2* carriers, only 4 had prior clinical testing and the percentage with syndromic cancer diagnoses was 47.8%, which is similar to published observations of clinically ascertained cases.^3^

The observed *BRCA1* to *BRCA2* case ratio of 1.0:1.8 is consistent with ratios in other relevant work.^29,30,31^ Previous research^3^ shows decreased penetrance for *BRCA2* carriers, which may be a contributing factor in the underascertainment of these cases compared with *BRCA1* cases; in our study, among those with prior testing and cancer diagnosis, more than 90% had *BRCA1*-associated cancer risk.

Among the 82.1% of *BRCA1/2* carriers who did not have prior clinical *BRCA1/2* testing, the return of genomic result was the start of a diagnostic process aimed at deciding if the risk variants were associated with current or past disease.^20^ For those with a personal history of *BRCA1/2*-associated cancer, a diagnosis of HBOC syndrome was now clear and the recommended management was changed to align with NCCN guidelines and to offer cascade testing for at-risk family members. Those without *BRCA1/2*-associated cancer at results disclosure did not have a diagnosis of HBOC syndrome, and long-term risk-reduction measures were recommended. For individuals with risk variants but without disease, the variants result was listed in their EHR problem list as a test result and not as an HBOC syndrome diagnosis.^20,32^ This distinction is important because continued surveillance is recommended, but syndrome diagnosis may not ever be achieved.^20,25,32^

Substantial infrastructure and health care practitioner collaboration were necessary to support the disclosure and follow-up care of the 183 participants whose biobank consent form allowed for clinical reporting.^32^ Consistent with the vision of learning health care systems,^33,34,35^ this disclosure and follow-up approach is being informed and improved by ongoing feedback from biobank participants, health care practitioners, and researchers. In addition, the disclosure can be used as a template for other settings where the genome-first care model is implemented.

Finally, optimal clinical management of patients identified through sequencing-based screening who do not otherwise meet the criteria for clinical testing is likely to evolve as more longitudinal outcomes data become available from this cohort and others. Given a near-term gap in evidence-based management, patients at Geisinger receive management based on previously developed protocols for *BRCA1/2*-positive individuals,^7^ but patients are informed that important knowledge gaps exist. The capture and aggregation of health outcomes data from this and other cohorts is essential to developing evidence that guides precision management strategies for individuals identified through whole exome sequencing–based screening.^36^ These health outcomes data, coupled with analysis of the cost of care (including cost of testing, counseling, and interventions), will ultimately determine the value of exome sequencing–based screening and inform the implementation of screening strategies for the *BRCA1/2* variants.

### Limitations

In general, the participants in the biobank were older than the health system’s patient population; more important, this study skewed toward those who received regular health care, increasing their opportunity to be enrolled in the biobank research. The higher incidence of breast cancer in the biobank population was likely a reflection of frequency of care and age; however, in our risk analysis, we used the biobank patients without evidence of *BRCA1/2* variants as the control population, which mitigated this bias.

The study still likely underestimated prevalence in this health care system–based cohort because of the (1) limitations to the current variant identification strategy (eg, our protocol did not include deletion-duplication analysis, which accounts for 10% of clinical variants)^3^; (2) absence of attempts to assign significance to missense variants beyond that established in ClinVar, and (3) estimated underrepresentation of cases with early-onset disease in the study cohort as a result of HBOC syndrome–associated mortality, given the age difference between the *BRCA1/2* cohort and the general health system population.^37^ The first 2 issues will be addressed in future work. We recognize that, because this study population is reflective of the local population and thus is overwhelmingly white,^16^ it may not be representative of other cohorts, particularly those from different racial/ethnic backgrounds. As with many areas of genomic medicine implementation, inclusion of greater numbers of individuals from underrepresented race/ethnicity is essential to a complete understanding of *BRCA1/2* risk.^38^ Data from other cohorts will be needed to clarify whether this *BRCA1* to *BRCA2* ratio finding is generalizable for population screening.

## Conclusions

The prevalence of *BRC1/2* variants in the general population may be substantially higher than was previously estimated, and reliance on personal and family history may be an inadequate measure to ascertain risk for *BRCA1/2* variants.

## Notes

Supplement. **eTable 1.** List of All Pathogenic and Likely Pathogenic Variants in *BRCA1* and *BRCA2* That Were Identified in Cohort **eTable 2.** The Overall Prevalence and Prevalence Controlling for Relatedness **eTable 3.** Cause of Death Analysis **eTable 4.** Relevant Cancer Among the Living Cases **eTable 5.** Table Demonstrating Groupings for Odds Ratio Calculations for Figure 2B-D **eFigure 1.** Pedigree and Case Story **eFigure 2.** Family Health History in EHR of *BRCA1/2* Cases (Women and Men)
