# Supplementary material for: Exome Sequencing–Based Screening for BRCA1/2 Expected Pathogenic Variants Among Adult Biobank Participants
Source: JAMA Netw Open. 2018 Sep 21;1(5):e182140. doi: 10.1001/jamanetworkopen.2018.2140 (PMC6324494; doi:10.1001/jamanetworkopen.2018.2140)
Supplement: Supplement. — eTable 1. List of All Pathogenic and Likely Pathogenic Variants in BRCA1 and BRCA2 That Were Identified in Cohort eTable 2. The Overall Prevalence and Prevalence Controlling for Relatedness eTable 3. Cause of Death Analysis eTable 4. Relevant Cancer Among the Living Cases eTable 5. Table Demonstrating Groupings for Odds Ratio Calculations for Figure 2B-D eFigure 1. Pedigree and Case Story eFigure 2. Family Health History in EHR of BRCA1/2 Cases (Women and Men) [file jamanetwopen-1-e182140-s001.pdf]

Supplementary Online Content

Manickam K, Buchanan AH, Schwartz MLB, et al. Exome sequencing–based screening for *BRCA1/2* expected pathogenic variants among adult biobank participants. *JAMA Netw Open.* 2018;1(5):e182140. doi:10.1001/jamanetworkopen.2018.2140

eTable 1. List of All Pathogenic and Likely Pathogenic Variants in *BRCA1* and *BRCA2* That Were Identified in Cohort

eTable 2. The Overall Prevalence and Prevalence Controlling for Relatedness

eTable 3. Cause of Death Analysis

eTable 4. Relevant Cancer Among the Living Cases

eTable 5. Table Demonstrating Groupings for Odds Ratio Calculations for Figure 2B-D

eFigure 1. Pedigree and Case Story

eFigure 2. Family Health History in EHR of *BRCA1/2* Cases (Women and Men)

This supplementary material has been provided by the authors to give readers additional information about their work.

©2018 Manickam K et al. *JAMA Network Open.*

eTable 1. List of All Pathogenic and Likely Pathogenic Variants in *BRCA1* and *BRCA2* That Were Identified in Cohort

eTable 1A. *BRCA1* (NM_007294.3) Variant List

	Gene	Function	cDNA position	Protein change	pLOF	LMM variant call	ClinVar Significance	ClinVar link (accessed 1/1/18)	# cases	Cancer Reported All	Cancer Reported Women
1	*BRCA1*	Frameshift	c.68_69delAG	p.Glu23Valfs*17	Yes	P	pathogenic *3	https://www.ncbi.nlm.nih.gov/clinvar/variation/17662/	5	1 of 5 (20%)	1 of 3 (33%)
2	*BRCA1*	Frameshift	c.143delT	p.Met48Serfs*2	Yes	P	pathogenic *3	https://www.ncbi.nlm.nih.gov/clinvar/variation/37412/	2	1 of 2 (50%)	1 of 1 (100%)
3	*BRCA1*	Missense	c.181T>G	p.Cys61Gly	No	P	pathogenic *3	https://www.ncbi.nlm.nih.gov/clinvar/variation/17661/	1	0 of 1 (0%)	0 of 1 (0%)
4	*BRCA1*	Splice variant	c.213-11T>G	NA	Yes	P	Pathogenic *2	http://www.ncbi.nlm.nih.gov/clinvar/variation/37449	1	1 of 1 (100%)	-
5	*BRCA1*	Splice variant	c.213-12A>G	NA	Yes	P	pathogenic *2	https://www.ncbi.nlm.nih.gov/clinvar/variation/37450/	1	0 of 1 (0%)	-
6	*BRCA1*	Frameshift	c.844_850dupTCATTAC	p.Gln284Leufs*5	Yes	P	pathogenic *3	https://www.ncbi.nlm.nih.gov/clinvar/variation/55735/	1	0 of 1 (0%)	-
7	*BRCA1*	Termination	c.1480C>T	p.Gln494*	Yes	P	pathogenic *3	https://www.ncbi.nlm.nih.gov/clinvar/variation/37415/	3	1 of 3 (33%)	1 of 2 (50%)
8	*BRCA1*	Frameshift	c.1812delA	p.Ala605Hisfs*7	Yes	P	pathogenic *3	https://www.ncbi.nlm.nih.gov/clinvar/variation/125513/	1	1 of 1 (100%)	1 of 1 (100%)
9	*BRCA1*	Frameshift	c.1881_1884delCAGT	p.Ser628Glufs*3	Yes	P	pathogenic *3	http://www.ncbi.nlm.nih.gov/clinvar/variation/54379	1	0 of 1 (0%)	0 of 1 (0%)
10	*BRCA1*	Frameshift	c.1953_1956delGAAA	p.Lys653Serfs*47	Yes	P	pathogenic *3	https://www.ncbi.nlm.nih.gov/clinvar/variation/37435/	2	1 of 2 (50%)	1 of 2 (50%)
11*	*BRCA1*	Frameshift	c.2090delT	p.Phe697Serfs*4	Yes	P	(novel)	https://www.ncbi.nlm.nih.gov/clinvar/variation/440454/	1	0 of 1 (0%)	0 of 1 (0%)
12	*BRCA1*	Termination	c.2309C>A	p.Ser770*	Yes	P	pathogenic *3	https://www.ncbi.nlm.nih.gov/clinvar/variation/54527/	8	4 of 8 (50%)	2 of 2 (100%)
13	*BRCA1*	Termination	c.2338C>T	p.Gln780*	Yes	P	pathogenic *3	https://www.ncbi.nlm.nih.gov/clinvar/variation/54540/	1	1 of 1 (100%)	1 of 1 (100%)
14	*BRCA1*	Frameshift	c.2443delA	p.Ile815Phefs*31	Yes	P	pathogenic *3	https://www.ncbi.nlm.nih.gov/clinvar/variation/54572/	1	0 of 1 (0%)	-
15	*BRCA1*	Frameshift	c.2457delC	p.Asp821Ilefs*25	Yes	P	pathogenic *3	https://www.ncbi.nlm.nih.gov/clinvar/variation/37471/	1	1 of 1 (100%)	1 of 1 (100%)
16	*BRCA1*	Frameshift	c.2679_2682delGAAA	p.Lys893Asnfs*160	Yes	P	pathogenic *3	https://www.ncbi.nlm.nih.gov/clinvar/variation/37481/	1	0 of 1 (0%)	-
17	*BRCA1*	Frameshift	c.2681_2682delAA	p.Lys894Thrfs*8	Yes	P	pathogenic *3	https://www.ncbi.nlm.nih.gov/clinvar/variation/17667/	1	0 of 1 (0%)	-
18	*BRCA1*	Termination	c.2722G>T	p.Glu908*	Yes	P	pathogenic *3	https://www.ncbi.nlm.nih.gov/clinvar/variation/54657/	3	2 of 3 (67%)	2 of 3 (66%)

©2018 Manickam K et al. *JAMA Network Open.*

	Gene	Function	cDNA position	Protein change	pLOF	LMM variant call	ClinVar Significance	ClinVar link (accessed 1/1/18)	# cases	Cancer Reported All	Cancer Reported Women
19	*BRCA1*	Frameshift	c.2823delT	p.Asn941Lysfs*59	Yes	P	pathogenic *3	https://www.ncbi.nlm.nih.gov/clinvar/variation/266306/	1	0 of 1 (0%)	0 of 1 (0%)
20	*BRCA1*	Frameshift	c.2882delA	p.Asn961Thrfs*39	Yes	P	pathogenic *3	https://www.ncbi.nlm.nih.gov/clinvar/variation/266315/	2	1 of 2 (50%)	1 of 1 (100%)
21	*BRCA1*	Frameshift	c.3005delA	p.Asn1002Thrfs*22	Yes	P	pathogenic *3	https://www.ncbi.nlm.nih.gov/clinvar/variation/17669/	1	1 of 1 (100%)	1 of 1 (100%)
22	*BRCA1*	Termination	c.3331C>T	p.Gln1111*	Yes	P	pathogenic *3	https://www.ncbi.nlm.nih.gov/clinvar/variation/54845/	3	1 of 3 (33%)	1 of 2 (50%)
23	*BRCA1*	Frameshift	c.3481_3491delGAAGAT ACTAG	p.Glu1161Phefs*3	Yes	P	pathogenic *3	https://www.ncbi.nlm.nih.gov/clinvar/variation/17684/	1	0 of 1 (0%)	-
24	*BRCA1*	Frameshift	c.3485delA	p.Asp1162Valfs*48	Yes	P	pathogenic *3	https://www.ncbi.nlm.nih.gov/clinvar/variation/37531/	1	0 of 1 (0%)	-
25	*BRCA1*	Termination	c.3607C>T	p.Arg1203*	Yes	P	pathogenic *3	https://www.ncbi.nlm.nih.gov/clinvar/variation/17671/	3	0 of 3 (0%)	0 of 2 (0%)
26	*BRCA1*	Frameshift	c.3648dupA	p.Ser1217Ilefs*2	Yes	P	pathogenic *3	https://www.ncbi.nlm.nih.gov/clinvar/variation/37535/	1	0 of 1 (0%)	0 of 1 (0%)
27	*BRCA1*	Frameshift	c.3700_3704delGTAAA	p.Val1234Glnfs*8	Yes	P	pathogenic *3	https://www.ncbi.nlm.nih.gov/clinvar/variation/37542/	1	0 of 1 (0%)	-
28	*BRCA1*	Termination	c.3718C>T	p.Gln1240*	Yes	P	pathogenic *3	https://www.ncbi.nlm.nih.gov/clinvar/variation/54978/	1	0 of 1 (0%)	-
29	*BRCA1*	Frameshift	c.3767_3768delCA	p.Thr1256Argfs*10	Yes	P	pathogenic *3	https://www.ncbi.nlm.nih.gov/clinvar/variation/182074/	2	1 of 2 (50%)	1 of 2 (50%)
30	*BRCA1*	Frameshift	c.4035delA	p.Glu1346Lysfs*20	Yes	P	pathogenic *3	https://www.ncbi.nlm.nih.gov/clinvar/variation/37560/	5	3 of 5 (60%)	3 of 5 (60%)
31	*BRCA1*	Termination	c.4222C>T	p.Gln1408*	Yes	P	pathogenic* 3	https://www.ncbi.nlm.nih.gov/clinvar/variation/55145/	1	0 of 1 (0%)	0 of 1 (0%)
32	*BRCA1*	Termination	c.4327C>T	p.Arg1443*	Yes	P	pathogenic *3	https://www.ncbi.nlm.nih.gov/clinvar/variation/17675/	1	0 of 1 (0%)	-
33	*BRCA1*	Termination	c.4389C>A	p.Tyr1463*	Yes	P	pathogenic *3	https://www.ncbi.nlm.nih.gov/clinvar/variation/55187/	1	0 of 1 (0%)	0 of 1 (0%)
34	*BRCA1*	Missense	c.4484G>T	p.Arg1495Met	No	P	pathogenic *2	https://www.ncbi.nlm.nih.gov/clinvar/variation/37598/	1	0 of 1 (0%)	-
35	*BRCA1*	Splice variant	c.4485-1G>T	NA	Yes	P	pathogenic *2	https://www.ncbi.nlm.nih.gov/clinvar/variation/246501/	2	1 of 2 (50%)	0 of 1 (0%)
36	*BRCA1*	Termination	c.4524G>A	p.Trp1508*	Yes	P	pathogenic *3	https://www.ncbi.nlm.nih.gov/clinvar/variation/55221/	1	0 of 1 (0%)	0 of 1 (0%)
37	*BRCA1*	Splice variant	c.4484+1G>A	NA	Yes	P	pathogenic *2	https://www.ncbi.nlm.nih.gov/clinvar/variation/37596/	1	0 of 1 (0%)	-
38	*BRCA1*	Termination	c.4689C>G	p.Tyr1563*	Yes	P	pathogenic *3	https://www.ncbi.nlm.nih.gov/clinvar/variation/37607/	1	1 of 1 (100%)	1 of 1 (100%)
39	*BRCA1*	Frameshift	c.4754_4755delCA	p.Pro1585Argfs*36	Yes	P	pathogenic *3	https://www.ncbi.nlm.nih.gov/clinvar/variation/55280/	1	0 of 1 (0%)	-

	Gene	Function	cDNA position	Protein change	pLOF	LMM variant call	ClinVar Significance	ClinVar link (accessed 1/1/18)	# cases	Cancer Reported All	Cancer Reported Women
40	*BRCA1*	Missense	c.4868C>G	p.Ala1623Gly	No	LP	conflicting *1	https://www.ncbi.nlm.nih.gov/clinvar/variation/37614/	1	0 of 1 (0%)	-
41	*BRCA1*	Missense	c.5066T>G	p.Met1689Arg	No	LP	conflicting *1	https://www.ncbi.nlm.nih.gov/clinvar/variation/37625/	2	1 of 2 (50%)	1 of 2 (50%)
42	*BRCA1*	Missense	c.5096G>A	p.Arg1699Gln	No	LP	conflicting *1	https://www.ncbi.nlm.nih.gov/clinvar/variation/37636/	1	0 of 1 (0%)	0 of 1 (0%)
43	*BRCA1*	Missense	c.5123C>A	p.Ala1708Glu	No	P	pathogenic *3	https://www.ncbi.nlm.nih.gov/clinvar/variation/55407/	1	1 of 1 (100%)	1 of 1 (100%)
44	*BRCA1*	Frameshift	c.5137delG	p.Val1713*	Yes	P	pathogenic *3	https://www.ncbi.nlm.nih.gov/clinvar/variation/55411/	1	0 of 1 (0%)	-
45	*BRCA1*	Splice variant	c.5153-2delA	NA	Yes	P	pathogenic *2	https://www.ncbi.nlm.nih.gov/clinvar/variation/55431/	1	0 of 1 (0%)	0 of 1 (0%)
46	*BRCA1*	Frameshift	c.5177_5180delGAAA	p.Arg1726Lysfs*3	Yes	P	pathogenic *3	https://www.ncbi.nlm.nih.gov/clinvar/variation/37644/	1	0 of 1 (0%)	-
47	*BRCA1*	Missense	c.5180G>A	p.Gly1706Glu	No	LP	pathogenic *3	https://www.ncbi.nlm.nih.gov/clinvar/variation/37638/	1	0 of 1 (0%)	-
48	*BRCA1*	Missense	c.5123C>A	p.Ala1708Glu	No	P	pathogenic *3	https://www.ncbi.nlm.nih.gov/clinvar/variation/55407/	1	1 of 1 (100%)	1 of 1 (100%)
49	*BRCA1*	Termination	c.5251C>T	p.Arg1751*	Yes	P	pathogenic *3	https://www.ncbi.nlm.nih.gov/clinvar/variation/55480/	1	0 of 1 (0%)	-
50	*BRCA1*	Frameshift	c.5266dupC	p.Gln1756Profs*74	Yes	P	pathogenic *3	https://www.ncbi.nlm.nih.gov/clinvar/variation/17677/	14	4 of 14 (29%)	2 of 5 (40%)
51	*BRCA1*	Termination	c.5345G>A	p.Trp1782*	Yes	P	pathogenic *3	https://www.ncbi.nlm.nih.gov/clinvar/variation/55544/	2	0 of 2 (0%)	0 of 2 (0%)
52	*BRCA1*	Termination	c.5503C>T	p.Arg1835*	Yes	P	pathogenic *3	https://www.ncbi.nlm.nih.gov/clinvar/variation/55601/	1	0 of 1 (0%)	-

* Row 11- this variant was first reported by this project in ClinVar (01-2017)

©2018 Manickam K et al. *JAMA Network Open.*

eTable 1B. *BRCA2* (NM_000059.3) Variant List

	Gene	Function	cDNA position	Protein change	pLOF	LMM variant call	ClinVar Significance	ClinVar link (accessed 6/7/17)	# cases	Cancer diagnosis All	Cancer diagnosis Women
1	*BRCA2*	Frameshift	c.22_23delAG	p.Arg8Alafs*5	Yes	P	pathogenic *3	https://www.ncbi.nlm.nih.gov/clinvar/variation/51267/	1	0 of 1 (0%)	-
2	*BRCA2*	Splice variant	c.517-2A>G	NA	Yes	P	path/likely path *2	https://www.ncbi.nlm.nih.gov/clinvar/variation/51801/	1	0 of 1 (0%)	0 of 1 (0%)
3	*BRCA2*	Frameshift	c.658_659delGT	p.Val220Ilefs*4	Yes	P	pathogenic *3	https://www.ncbi.nlm.nih.gov/clinvar/variation/9342/	2	0 of 2 (0%)	0 of 1 (0%)
4	*BRCA2*	Frameshift	c.1189_1190insTTAG	p.Gln397Leufs*25	Yes	P	pathogenic*3	https://www.ncbi.nlm.nih.gov/clinvar/variation/51080/	2	0 of 2 (0%)	-
5	*BRCA2*	Frameshift	c.1192delC	p.Leu398*	Yes	P	pathogenic*2	https://www.ncbi.nlm.nih.gov/clinvar/variation/409454/	1	0 of 1 (0%)	-
6	*BRCA2*	Frameshift	c.1321_1324delACTT	p.Thr441Glnfs*18	Yes	P	pathogenic*1	https://www.ncbi.nlm.nih.gov/clinvar/variation/418992/	1	1 of 1 (100%)	1 of 1 (100%)
7 *	*BRCA2*	Termination	c.1381G>T	p.Glu461*	Yes	P	(novel)	https://www.ncbi.nlm.nih.gov/clinvar/variation/440457/	1	0 of 1 (0%)	0 of 1 (0%)
8	*BRCA2*	Frameshift	c.1763_1766delATAA	p.Asn588Serfs*25	Yes	P	pathogenic *3	https://www.ncbi.nlm.nih.gov/clinvar/variation/51187/	1	1 of 1 (100%)	1 of 1 (100%)
9	*BRCA2*	Termination	c.1800T>G	p.Tyr600*	Yes	P	pathogenic *3	https://www.ncbi.nlm.nih.gov/clinvar/variation/51199/	1	0 of 1 (0%)	0 of 1 (0%)
10	*BRCA2*	Frameshift	c.1813dupA	p.Ile605Asnfs*11	Yes	P	pathogenic *3	https://www.ncbi.nlm.nih.gov/clinvar/variation/37762/	3	1 of 3 (33%)	1 of 3 (33%)
11	*BRCA2*	Termination	c.1832C>G	p.Ser611*	Yes	P	pathogenic *3	https://www.ncbi.nlm.nih.gov/clinvar/variation/228323/	1	0 of 1 (0%)	-
12	*BRCA2*	Frameshift	c.1929delG	p.Arg645Glufs*15	Yes	P	pathogenic *3	https://www.ncbi.nlm.nih.gov/clinvar/variation/37769/	2	1 of 2 (50%)	1 of 1 (100%)
13	*BRCA2*	Frameshift	c.2092delC	p.Leu698Tyrfs*32	Yes	P	pathogenic *3	https://www.ncbi.nlm.nih.gov/clinvar/variation/37774/	1	0 of 1 (0%)	0 of 1 (0%)
14	*BRCA2*	Termination	c.2339C>G	p.Ser780*	Yes	P	pathogenic *3	https://www.ncbi.nlm.nih.gov/clinvar/variation/141070/	1	0 of 1 (0%)	-
15	*BRCA2*	Termination	c.2368G>T	p.Glu790*	Yes	P	pathogenic *3	https://www.ncbi.nlm.nih.gov/clinvar/variation/91776/	1	0 of 1 (0%)	0 of 1 (0%)
16	*BRCA2*	Termination	c.2409T>G	p.Tyr803*	Yes	P	pathogenic *3	https://www.ncbi.nlm.nih.gov/clinvar/variation/37784/	1	0 of 1 (0%)	0 of 1 (0%)
17	*BRCA2*	Frameshift	c.2808_2811delACAA	p.Ala938Profs*21	Yes	P	pathogenic *3	https://www.ncbi.nlm.nih.gov/clinvar/variation/9322/	2	1 of 2 (50%)	1 of 1 (100%)
18	*BRCA2*	Frameshift	c.3167_3170delAAAA	p.Gln1056Argfs*3	Yes	P	pathogenic *3	https://www.ncbi.nlm.nih.gov/clinvar/variation/51413/	1	0 of 1 (0%)	0 of 1 (0%)
19	*BRCA2*	Termination	c.3172A>T	p.Lys1058*	Yes	P	pathogenic *3	https://www.ncbi.nlm.nih.gov/clinvar/variation/182196/	2	0 of 2 (0%)	0 of 1 (0%)
20	*BRCA2*	Termination	c.3187C>T	p.Gln1063*	Yes	P	pathogenic *3	https://www.ncbi.nlm.nih.gov/clinvar/variation/228324/	10	0 of 10 (0%)	0 of 6 (0%)

©2018 Manickam K et al. *JAMA Network Open.*

	Gene	Function	cDNA position	Protein change	pLOF	LMM variant call	ClinVar Significance	ClinVar link (accessed 1/1/18)	# cases	Cancer Reported All	Cancer Reported Women
21	*BRCA2*	Frameshift	c.3545_3546delTT	p.Phe1182*	Yes	P	pathogenic *3	https://www.ncbi.nlm.nih.gov/clinvar/variation/37846/	6	0 of 6 (0%)	0 of 2 (0%)
22	*BRCA2*	Frameshift	c.3680_3681delTG	p.Leu1227Glnfs*5	Yes	P	pathogenic *3	https://www.ncbi.nlm.nih.gov/clinvar/variation/51504/	1	0 of 1 (0%)	-
23	*BRCA2*	Frameshift	c.3847_3848delGT	p.Val1283Lysfs*2	Yes	P	pathogenic *3	https://www.ncbi.nlm.nih.gov/clinvar/variation/37859/	5	2 of 5 (40%)	1 of 3 (33%)
24 *	*BRCA2*	Frameshift	c.4103delT	p.Leu1368Tyrfs*6	Yes	P	(novel)	https://www.ncbi.nlm.nih.gov/clinvar/variation/440456/	1	0 of 1 (0%)	0 of 1 (0%)
25	*BRCA2*	Frameshift	c.4284dupT	p.Gln1429Serfs*9	Yes	P	pathogenic *3	https://www.ncbi.nlm.nih.gov/clinvar/variation/37892/	2	0 of 2 (0%)	0 of 2 (0%)
26	*BRCA2*	Frameshift	c.4419delC	p.Asn1473Lysfs*6	Yes	P	Pathogenic *1	https://www.ncbi.nlm.nih.gov/clinvar/variation/420191/	1	0 of 1 (0%)	0 of 1 (0%)
27	*BRCA2*	Termination	c.4588A>T	p.Lys1530*	Yes	P	pathogenic *3	https://www.ncbi.nlm.nih.gov/clinvar/variation/51678/	1	0 of 1 (0%)	0 of 1 (0%)
28	*BRCA2*	Frameshift	c.4876_4877delAA	p.Asn1626Serfs*12	Yes	P	pathogenic *3	https://www.ncbi.nlm.nih.gov/clinvar/variation/37929/	2	0 of 2 (0%)	0 of 1 (0%)
29	*BRCA2*	Termination	c.4889C>G	p.Ser1630*	Yes	P	Pathogenic *3	https://www.ncbi.nlm.nih.gov/clinvar/variation/51732/	1	0 of 1 (0%)	0 of 1 (0%)
30	*BRCA2*	Frameshift	c.4936_4939delGAAA	p.Glu1646Glnfs*23	Yes	P	pathogenic *3	https://www.ncbi.nlm.nih.gov/clinvar/variation/37935/	1	0 of 1 (0%)	0 of 1 (0%)
31	*BRCA2*	Termination	c.4965C>G	p.Tyr1655*	Yes	P	pathogenic *3	https://www.ncbi.nlm.nih.gov/clinvar/variation/37936/	14	1 of 14 (7%)	1 of 8 (12%)
32	*BRCA2*	Frameshift	c.4947_4948delAA	p.Pro1651Cysfs*14	Yes	P	Pathogenic* 3	https://www.ncbi.nlm.nih.gov/clinvar/variation/51747/	1	0 of 1 (0%)	0 of 1 (0%)
33	*BRCA2*	Frameshift	c.5073dupA	p.Trp1692Metfs*3	Yes	P	pathogenic *3	https://www.ncbi.nlm.nih.gov/clinvar/variation/37943/	2	0 of 2 (0%)	-
34	*BRCA2*	Frameshift	c.5157_5161delTTCAA	p.Asn1719Lysfs*6	Yes	P	pathogenic *3	https://www.ncbi.nlm.nih.gov/clinvar/variation/51780/	3	1 of 3 (33%)	-
35	*BRCA2*	Frameshift	c.5217_5220delTTTA	p.Tyr1739*	Yes	P	pathogenic *3	https://www.ncbi.nlm.nih.gov/clinvar/variation/51820/	1	0 of 1 (0%)	-
36	*BRCA2*	Frameshift	c.5350_5351delAA	p.Asn1784Hisfs*2	Yes	P	pathogenic *3	https://www.ncbi.nlm.nih.gov/clinvar/variation/37959/	2	1 of 2 (50%)	1 of 1 (100%)
37 *	*BRCA2*	Frameshift	c.5352dupC	p.Thr1785Hisfs*2	Yes	P	(novel)	https://www.ncbi.nlm.nih.gov/clinvar/variation/548420/	2	1 of 2 (50%)	1 of 1 (100%)
38	*BRCA2*	Frameshift	c.5410_5411delGT	p.Val1804Lysfs*2	Yes	P	pathogenic *3	https://www.ncbi.nlm.nih.gov/clinvar/variation/37963/	1	0 of 1 (0%)	-
39	*BRCA2*	Frameshift	c.5576_5579delTTAA	p.Ile1859Lysfs*3	Yes	P	pathogenic *3	https://www.ncbi.nlm.nih.gov/clinvar/variation/37975/	1	0 of 1 (0%)	-
40	*BRCA2*	Termination	c.5614A>T	p.Lys1872*	Yes	P	pathogenic *3	https://www.ncbi.nlm.nih.gov/clinvar/variation/37979/	1	0 of 1 (0%)	0 of 1 (0%)
41	*BRCA2*	Frameshift	c.5681dupA	p.Tyr1894*	Yes	P	pathogenic *3	https://www.ncbi.nlm.nih.gov/clinvar/variation/37988/	1	0 of 1 (0%)	0 of 1 (0%)

©2018 Manickam K et al. *JAMA Network Open.*

	Gene	Function	cDNA position	Protein change	pLOF	LMM variant call	ClinVar Significance	ClinVar link (accessed 1/1/18)	# cases	Cancer Reported All	Cancer Reported Women
42	*BRCA2*	Frameshift	c.5722_5723delCT	p.Leu1908Argfs*2	Yes	P	pathogenic *3	https://www.ncbi.nlm.nih.gov/clinvar/variation/9320/	24	7 of 24 (29%)	5 of 15 (33%)
43	*BRCA2*	Termination	c.5857G>T	p.Glu1953*	Yes	P	pathogenic *3	https://www.ncbi.nlm.nih.gov/clinvar/variation/51952/	1	0 of 1 (0%)	0 of 1 (0%)
44	*BRCA2*	Frameshift	c.5946delT	p.Ser1982Argfs*22	Yes	P	pathogenic *3	https://www.ncbi.nlm.nih.gov/clinvar/variation/9325/	3	0 of 3 (0%)	0 of 1 (0%)
45	*BRCA2*	Termination	c.6037A>T	p.Lys2013*	Yes	P	pathogenic *3	https://www.ncbi.nlm.nih.gov/clinvar/variation/38016/	5	0 of 5 (0%)	0 of 1 (0%)
46	*BRCA2*	Frameshift	c.6405_6409delCTTAA	p.Asn2135Lysfs*3	Yes	P	pathogenic *3	https://www.ncbi.nlm.nih.gov/clinvar/variation/38043/	1	0 of 1 (0%)	0 of 1 (0%)
47	*BRCA2*	Frameshift	c.6468_6469delTC	p.Gln2157Ilefs*18	Yes	P	pathogenic *3	https://www.ncbi.nlm.nih.gov/clinvar/variation/38047/	1	0 of 1 (0%)	-
48	*BRCA2*	Frameshift	c.6644_6647delACTC	p.Tyr2215Serfs*13	Yes	P	pathogenic *3	https://www.ncbi.nlm.nih.gov/clinvar/variation/38060/	3	0 of 3 (0%)	0 of 3 (0%)
49	*BRCA2*	Frameshift	c.6998dupT	p.Pro2334Thrfs*6	Yes	P	pathogenic *3	https://www.ncbi.nlm.nih.gov/clinvar/variation/219496/	1	0 of 1 (0%)	-
50	*BRCA2*	Frameshift	c.7069_7070delCT	p.Leu2357Valfs*2	Yes	P	pathogenic *3	https://www.ncbi.nlm.nih.gov/clinvar/variation/38082/	18	2 of 18 (11%)	1 of 12 (8%)
51	*BRCA2*	Splice variant	c.7436-2A>T	NA	Yes	P	pathogenic *1	http://www.ncbi.nlm.nih.gov/clinvar/variation/52330	1	0 of 1 (0%)	-
52	*BRCA2*	Termination	c.7558C>T	p.Arg2520*	Yes	P	pathogenic *3	https://www.ncbi.nlm.nih.gov/clinvar/variation/52353/	2	1 of 2 (50%)	1 of 2 (50%)
53	*BRCA2*	Frameshift	c.7762delA	p.Ile2588Tyrfs*60	Yes	P	pathogenic *3	https://www.ncbi.nlm.nih.gov/clinvar/variation/52405/	2	1 of 2 (50%)	0 of 1 (0%)
54	*BRCA2*	Missense	c.7878G>C	p.Trp2626Cys	No	P	pathogenic *3	https://www.ncbi.nlm.nih.gov/clinvar/variation/38125/	2	0 of 2 (0%)	0 of 1 (0%)
55	*BRCA2*	Missense	c.8243G>A	p.Gly2748Asp	No	P	pathogenic *3	https://www.ncbi.nlm.nih.gov/clinvar/variation/52535/	1	0 of 1 (0%)	0 of 1 (0%)
56	*BRCA2*	Splice variant	c.8487+1G>A	NA	Yes	P	pathogenic *3	https://www.ncbi.nlm.nih.gov/clinvar/variation/52602/	3	1 of 3 (33%)	0 of 1 (0%)
57	*BRCA2*	Termination	c.8489G>A	p.Trp2830*	Yes	P	pathogenic *3	https://www.ncbi.nlm.nih.gov/clinvar/variation/52605/	2	0 of 2 (0%)	-
58	*BRCA2*	Frameshift	c.8537_8538delAG	p.Glu2846Glyfs*22	Yes	P	pathogenic *3	https://www.ncbi.nlm.nih.gov/clinvar/variation/9328/	1	0 of 1 (0%)	-
59	*BRCA2*	Splice variant	c.8755-1G>A	NA	Yes	P	Uncertain significance *3	https://www.ncbi.nlm.nih.gov/clinvar/variation/38183/	1	0 of 1 (0%)	-
60	*BRCA2*	Frameshift	c.8904delC	p.Thr2968Cysfs*7	Yes	P	pathogenic *3	https://www.ncbi.nlm.nih.gov/clinvar/variation/38192/	4	2 of 4 (50%)	2 of 3 (66%)
61	*BRCA2*	Splice variant	c.8953+1G>T	NA	Yes	P	pathogenic *3	https://www.ncbi.nlm.nih.gov/clinvar/variation/38198/	3	0 of 3 (0%)	0 of 1 (0%)
62	*BRCA2*	Missense	c.9004G>A	p.Glu3002Lys	No	P	conflicting *1	https://www.ncbi.nlm.nih.gov/clinvar/variation/38201/	1	0 of 1 (0%)	-

©2018 Manickam K et al. *JAMA Network Open.*

	Gene	Function	cDNA position	Protein change	pLOF	LMM variant call	ClinVar Significance	ClinVar link (accessed 1/1/18)	# cases	Cancer Reported All	Cancer Reported Women
63	*BRCA2*	Frameshift	c.9097dupA	p.Thr3033Asnfs*11	Yes	P	pathogenic *3	https://www.ncbi.nlm.nih.gov/clinvar/variation/38208/	1	0 of 1 (0%)	0 of 1 (0%)
64	*BRCA2*	Frameshift	c.9253delA	P.Thr3085Glnfs*19	Yes	P	pathogenic *3	https://www.ncbi.nlm.nih.gov/clinvar/variation/52785/	3	1 of 3 (33%)	1 of 2 (50%)
65	*BRCA2*	Frameshift	c.9672dupA	p.Tyr3225Ilefs*30	Yes	P	pathogenic *3	https://www.ncbi.nlm.nih.gov/clinvar/variation/126217/	1	0 of 1 (0%)	-
66 *	*BRCA2*	Frameshift	c.9777delT	p.Ile3259Metfs*16	Yes	LP	(novel)	NA	1	0 of 1 (0%)	0 of 1 (0%)

* Row 7- this variant was first reported by this project in ClinVar (01-2017)

* Row 24 - this variant was first reported by this project in ClinVar (12-2016)

* Row 37 – this variant was first reported by this project in ClinVar (08-2017)

* Row 66 - report of this variant in ClinVar by this project is pending

©2018 Manickam K et al. *JAMA Network Open.*

eTable 2. The Overall Prevalence and Prevalence Controlling for Relatedness

The overall prevalence in the entire cohort (N=50,726) and the prevalence in a subcohort (N=38,339) where only one individual in every first and second degree relationship was included. The algorithm for determining relatedness has been described (Ref 19, 25)

**COHORT** N	**CASES** N (%)	**PREVALENCE**
50726	267 (0.53)	1/190
38339	213 (0.56)	1/180

©2018 Manickam K et al. *JAMA Network Open.*

eTable 3. Cause of Death Analysis

Among the 23 cases of individuals with P/LP *BRCA1/2* variants there were 11 (47.8%) cases with relevant cancers, and at least 9 (39.1%) died of HBOC associated disease.

		**Cause of Death**			**Family History (EHR based)**		**HBOC Associated cancers**				
	N=23	BRCA associated (N=9)	Non-BRCA Associated (N=11)	Unknown (N=3)	Family History (+) (N=10)	Family History (-) (N=13)	Breast	Ovarian	Prostate	Pancreatic	None
**Female**	12	7	3	2	8	4	4	5	-	0	3
**Male**	11	2	8	1	2	9	0	-	1	1	9
BRCA1	9	4	4	1	5	4	1	3	0	0	5
BRCA2	14	5	7	2	5	9	3	2	1	1	7
**Prior Testing**	4	4	0	0	4	0	2	2	0	0	0
**No Prior Testing**	19	5	11	3	6	13	2	3	1	1	12

©2018 Manickam K et al. *JAMA Network Open.*

eTable 4. Relevant Cancer Among the Living Cases

18.4% (45/244) of living *BRCA1/2* cases were found to have relevant cancer diagnoses. The cancers are broken down by anatomic sites in women v. men, BRCA1 v. BRCA2, and in those with and without prior testing.

		**Family History (EHR based)**		**HBOC Associated cancers**				
	N=244	Family History (+) (N=129)	Family History (-) (N=115)	Breast (N=27)	Ovarian N=10	Prostate (N=9)	Melanoma (N=2)	None (N=199)
**Female** *	136	91	45	27 *	10 *	-	0	102
**Male**	108	38	70	0	-	9	2	97
BRCA1	87	51	36	14	7	4	2	61
BRCA2	157	78	79	13	3	5	0	138
**Prior Testing**	44	38	6	11	4	1	0	30
**No Prior Testing**	200	91	109	16	6	8	2	169

* three cases of women wih breast and ovarian cancer

©2018 Manickam K et al. *JAMA Network Open.*

eTable 5. Table Demonstrating Groupings for Odds Ratio Calculations for Figure 2B-D

	Cases<br>Pos P/LP	Controls<br>Neg P/LP
Cancer	a	b
No cancer	c	d
$\frac{a/c}{b/d}$		

©2018 Manickam K et al. *JAMA Network Open.*

eFigure 1. Pedigree and Case Story

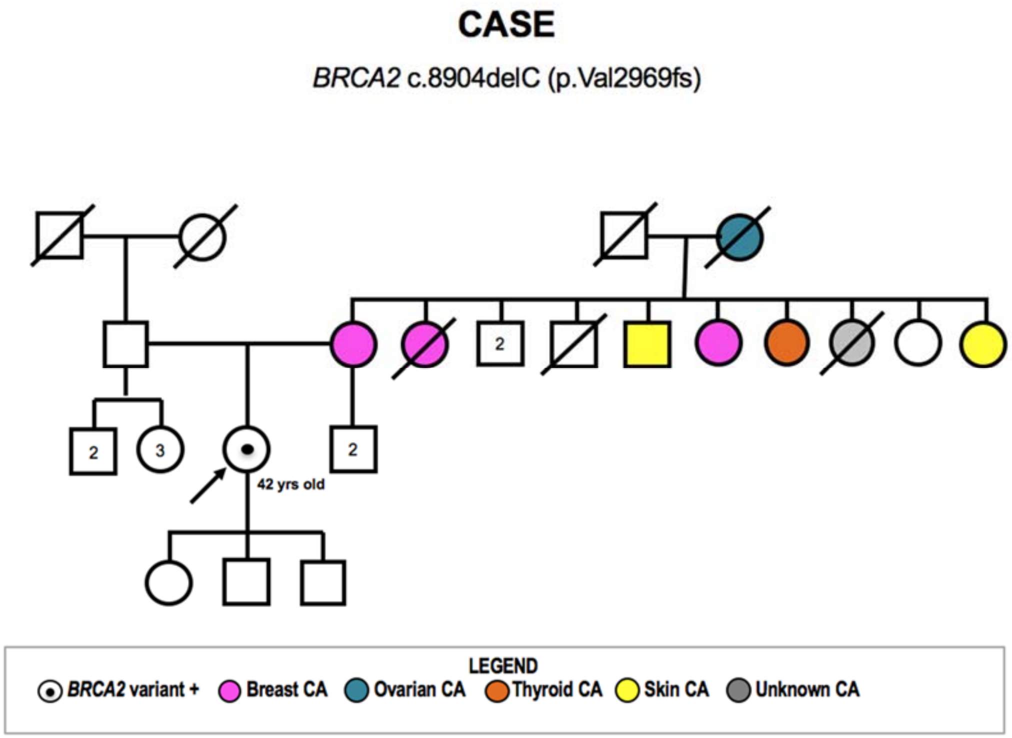

42-year-old woman with new diagnosis of bilateral ductal carcinoma in situ (DCIS). A woman with no previous cancer diagnosis received notification of a pathogenic *BRCA2* variant (c.8904delC - ClinVar 38192). Her family history is significant for the following HBOC related cancers: mother diagnosed with breast cancer at age 54 (living), maternal aunt diagnosed with breast cancer at age 39 (deceased), a second maternal aunt diagnosed with breast cancer at age 38 (living), and maternal grandmother diagnosed with ovarian cancer at age 38 (deceased). She had a mammogram that was previously recommended but never carried out. A left breast abnormality on this screening mammogram prompted a biopsy, leading to a diagnosis of DCIS. Subsequent bilateral breast MRI showed no further abnormalities in either breast. She proceeded to bilateral mastectomies, with pathology showing residual DCIS in the left breast and an occult DCIS in the right breast. She was thereby the fourth case of a "group 3" diagnosis (see Table 2) in addition to the three cases described in Buchanan et al.[21]

©2018 Manickam K et al. *JAMA Network Open.*

eFigure 2. Family Health History in EHR of *BRCA1/2* Cases (Women and Men)

Panel A (top). Data extraction from the pre-return of results EHR of all BRCA1/2 carriers.

Panel B (bottom). Data extraction from the post-return of results EHR of a subset of BRCA1/2 carriers who reviewed Family Health History with a genetic counselor.

Note: In both settings there were twice as many men with no recorded family history of relevant disease in their EHR compared to women.

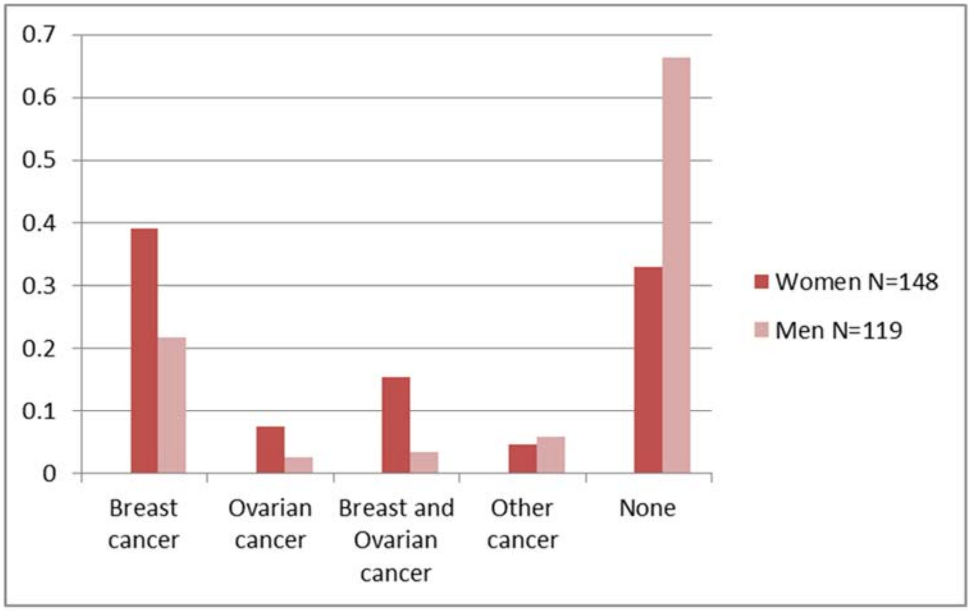

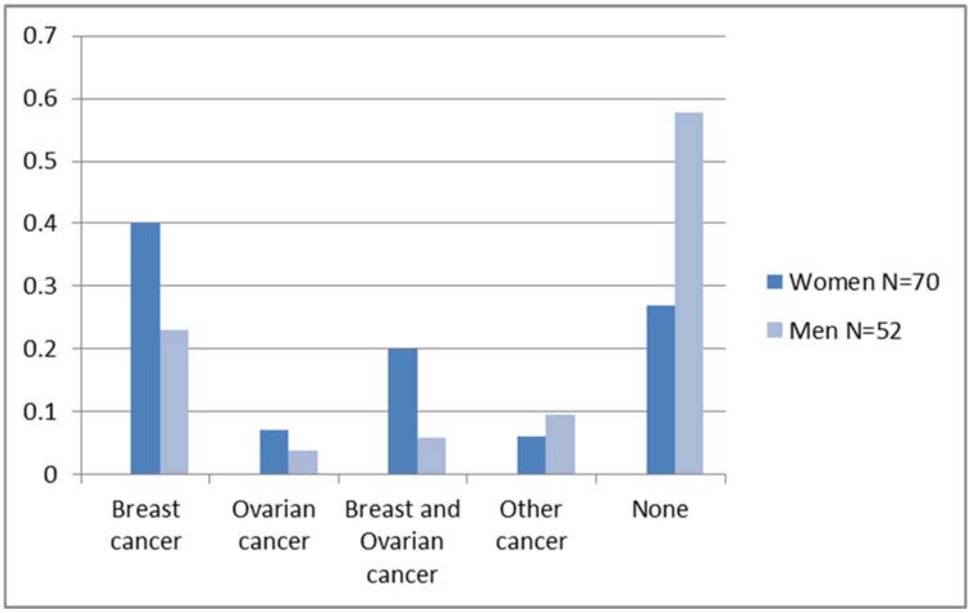

©2018 Manickam K et al. *JAMA Network Open.*
